# Case Report: Successful Intravenous Lipid Emulsion Therapy for Canine Amphetamine Toxicosis

**DOI:** 10.3389/fvets.2022.938021

**Published:** 2022-07-12

**Authors:** Stephanie Harris, Maureen A. McMichael, Roy Harmon, Dawn Boothe

**Affiliations:** ^1^Department of Clinical Sciences, Auburn University College of Veterinary Medicine, Auburn, AL, United States; ^2^Department of Anatomy, Physiology and Pharmacology, Auburn University College of Veterinary Medicine, Auburn, AL, United States

**Keywords:** Adderall XR^Ⓡ^, overdose, methamphetamine, poisoning, intralipid, dog

## Abstract

A 4-year-old, female-spayed, mixed breed dog, weighing 24.2 kg, was presented for acute ingestion of ~12.3 mg/kg of Adderall XR^Ⓡ^, an extended-release amphetamine medication. In dogs, the oral median lethal dose for amphetamines ranges anywhere from 9–11 mg/kg to 20–27 mg/kg. On presentation, the patient was agitated, tachycardic and hypertensive. Initial treatment was instituted with intravenous lipid emulsion (IVLE) therapy, and baseline and post-treatment amphetamine concentrations were quantified in serum and plasma. In both serum and plasma, post-IVLE concentrations of amphetamine were lower 1 h after treatment and IVLE was the only treatment instituted during this time. The dog improved significantly while in hospital and was discharged <24 h after presentation. This is the first known reported use of IVLE for treatment of amphetamine toxicosis with documented decreases in both serum and plasma amphetamine levels shortly after administration of IVLE.

## Introduction

In humans, a variety of brand name and generic versions of amphetamines are commonly prescribed for attention deficit hyperactivity disorder (ADHD), narcolepsy and weight loss. Amphetamine is also a DEA schedule II-controlled substance and has a high potential for abuse. It is used off-label by college students and young adults for memory enhancement, as a study agent and recreationally. Both immediate and sustained-release products are used for these purposes (1, 2).

Due to the frequent use of amphetamines, it is not uncommon to see accidental ingestion and toxicosis in dogs (2, 3). In both humans and dogs, amphetamine toxicosis causes a variety of clinical signs, including hyperthermia, agitation, arrhythmias and in large doses, seizures, and death. There is no antidote for amphetamine toxicosis, and the mainstay of treatment includes supportive care and management of clinical signs. It is noteworthy that amphetamines are highly lipophilic molecules and intravenous lipid emulsion (IVLE) therapy may be indicated in life-threatening amphetamine toxicosis (4).

Intravenous lipid emulsion is a relatively inexpensive treatment that was originally formulated as a component of parenteral nutrition, but is also used as a treatment of certain toxic agents that are lipophilic. The complete mechanism of how IVLE works with lipophilic agents is not fully understood and there are several theories describing the mechanism of action, with the primary two being the “lipid sink” and “lipid shuttle” theories. Both theories describe accelerated clearance of the lipid-bound toxin from the body and redirection of the toxin from sensitive organs such as the brain and heart (5). IVLE has a low incidence of adverse side effects when used according to recommended guidelines (6, 7). Side effects reported in humans include lipid embolism, anaphylactic reaction, fat overload syndrome, coagulation issues and pancreatitis (6).

Treatment of amphetamine toxicosis in both human and veterinary medicine is mainly supportive, with focus on management of life-threatening arrhythmias, seizure control, and temperature reduction (2, 3). If the ingestion is caught early and the patient is asymptomatic, gastric decontamination can be performed with emesis or gastric lavage, followed by activated charcoal. Prognosis depends on severity and duration of clinical signs at presentation. If the patient did not ingest a lethal dose and is treated early with appropriate decontamination and if warranted, sedation, anticonvulsants and anti-arrythmic agents, prognosis for full recovery is good (1–3).

## Case Description

A 4-year-old, female-spayed mixed breed canine weighing 24.2 kg was presented to the emergency service of a veterinary teaching hospital for ingestion of up to 12.3 mg/kg of an oral formulation of Adderall XR^Ⓡ^ within 3 h prior of presentation. The owners reported finding several tablets partially chewed and strewn about and the dog was hypersalivating and acting anxious. The dog was reportedly otherwise healthy and not on any concurrent medications.

Upon physical examination, the dog was agitated with hyperemic sclera and mucous membranes, had mydriasis bilaterally and was tachycardic with a heart rate of 160 beats per minute, and had bounding, synchronous pulses. She was hypertensive with a systolic blood pressure of 190 mm Hg and was hypersalivating with a respiratory rate of 48 breaths per minute. No tremors, seizures or hyperthermia (38.6°C) were noted on presentation.

An IV catheter was placed, and blood was collected for a venous electrolyte and blood gas profile, which showed a sodium of 138.6 mmol/L (141.8–148.6 mmol/L) and chloride of 121.0 mmol/L (111.7–118.3 mmol/L), with the remainder of her values within the laboratory's reference interval. Blood was also obtained at this time for measurement of serum and plasma amphetamine concentrations and was stored at −80°C.

Due to the reported dosage of ingested amphetamine with concurrent clinical signs, IVLE therapy was administered upon presentation through a peripheral venous catheter. An initial bolus of 1.5 ml/kg of IVLE was administered over 5 min, followed by a 0.25 ml/kg/min CRI over 1 h. One hour after completion of IVLE treatment, blood samples were drawn again for repeated measurement of amphetamine concentrations and stored similarly. Immediately after administration of IVLE, the patient's Doppler blood pressure remained elevated at 200 mm Hg and the heart rate was 88 beats per minute. No salivation or tremors were noted at that time. One hour after completion of IVLE administration, at time of sample collection, the Doppler blood pressure had decreased to 156 mm Hg and the heart rate was 124 beats per minute. Clinically the patient was more quiet and was resting.

After collection of blood 1 h after IVLE administration, the dog was started on a balanced crystalloid solution (Ph-lyte) at 90 ml/h and a single dose of acepromazine at 0.05 mg/kg was administered IV due to perceived anxiety. The patient ate well soon after and was administered a single oral dose of 1 g/kg of activated charcoal with sorbitol. Five hours after presentation, the patient was noted to have some occasional muscle tremors, excitement, and agitation and was also noted licking her lips. Due to these signs, a second dose of 0.05 mg/kg acepromazine was administered IV, along with maropitant at 1 mg/kg IV due to perceived nausea. By morning, ~10 h after presentation, the patient was asymptomatic with normal heart rate and blood pressure parameters. She was discharged later that day, <24 h after presentation, with 4 mg/kg oral trazodone as needed for potential recurrence of agitation.

## Methods/Measurements of Samples

Blood samples were drawn from the patient immediately prior to IVLE therapy and 1 h after IVLE therapy was completed. The samples were placed into three separate blood collection tubes (heparin, EDTA, serum tube with no additives). Within 1 h of collection, the samples underwent centrifugation and the plasma and serum were collected individually and stored at −80°C until time of analysis.

**Figure 1:**
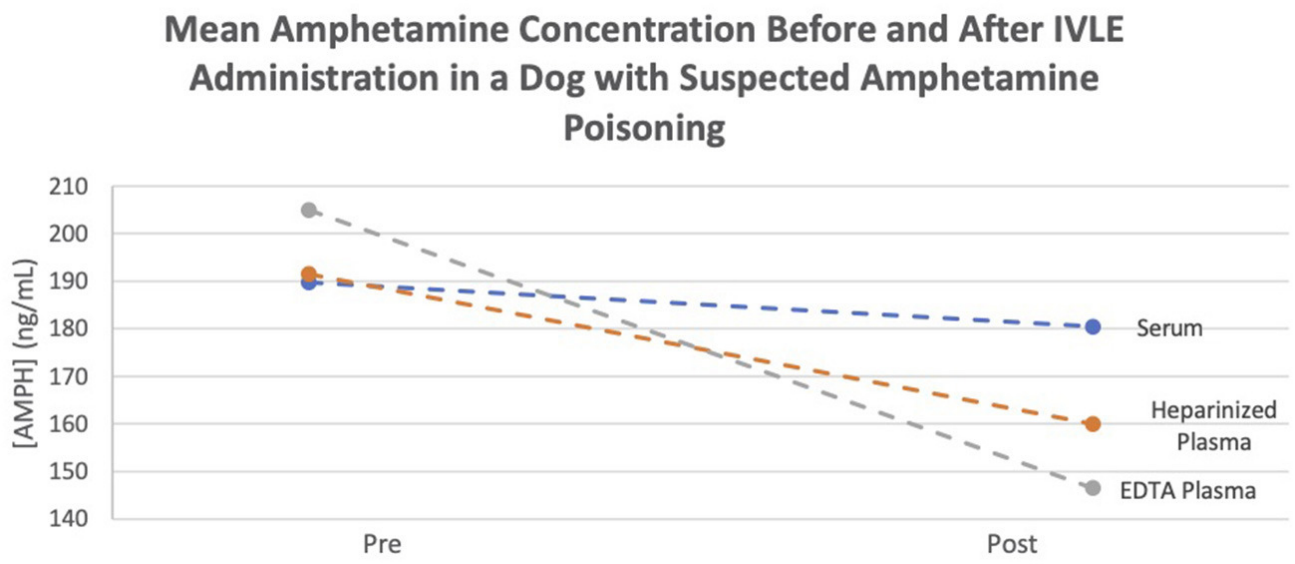
Mean amphetamine concentration before and after IVLE administration in a dog with suspected amphetamine poisoning.

Amphetamine levels were quantified using a Syva® Emit® II Plus immunoassay with a Siemens Dimension Xpand Plus automated analyzer. The assay was validated by the institution's Clinical Pharmacology Laboratory prior to sample analysis. The limit of quantitation for amphetamine was 10 ng/ml. The recovery (±CV) for amphetamine plasma control samples with concentrations of 300 and 600 ng/ml was 104.1 ± 5.9 and 105.1 ± 8.0%, respectively. Assays were performed on three consecutive days with single assays being performed on the first 2 days and two assays performed on the third day. The average readings of all pre- and post-samples are documented in Table 1. All samples were shown to have an average decrease in amphetamine concentrations 1 h after IVLE administration (Figure 1).

**Table 1:** Mean amphetamine values in a dog with suspected amphetamine poisoning before and after IVLE administration.

	**Serum**	**Heparinized plasma**	**EDTA plasma**
Pre-amphetamine concentration (ng/ml)	189.8	191.5	205.0
Post-amphetamine concentration (ng/ml)	180.5	160.0	146.5

## Discussion

Intravenous lipid emulsion therapy has antidotal effects that vary with the lipophilicity of the toxic agent: the more lipophilic the agent, the more effective IVLE is thought to be. The lipophilicity of a drug and thus its solubility and extent of distribution determine its log *P* value, where *P* represents the partition co-efficient. The higher the log *P* value, the more lipophilic the drug, with log *P* values >1 representing lipid soluble agents (4, 8, 9). The log *P* of amphetamine is 1.76 (4, 8).

Hypothesized mechanisms of action for IVLE therapy are thought to be in part due to a “lipid sink” effect, wherein the toxic agent is sequestered in a lipid compartment within the intravascular space and then eliminated. A “lipid shuttle” theory is also commonly described, that describes the back-and-forth movement of the toxin-bound emulsified lipid, thereby diverting the toxin from sensitive organs such as the heart and brain. This redirects the toxin-bound lipid to organs such as the liver, hastening its metabolism. Other proposed theories include the utilization of free fatty acids as an energy source by the myocardium, restoration of the mitochondrial function, an increase in intracellular calcium, and reduction of nitric oxide and vasodilatation (5, 8, 9).

The use of IVLE in the treatment of lipid-soluble toxins has been postulated to be effective at increasing the rate of drug elimination and decreasing adverse effects. In humans, the use of IVLE is generally reserved for severe toxicosis and life-threatening conditions, which differs from the approach in veterinary medicine, where administration of IVLE is generally initiated earlier during therapy in symptomatic patients (9, 10). The use of IVLE in veterinary medicine is warranted for toxicoses associated with a high morbidity, for which there is no antidote, and particularly where traditional therapies have failed or are cost prohibitive. In both human and veterinary medicine, IVLE is considered relatively safe and adverse reactions are rare, consisting of either an anaphylactoid reaction to the lipids themselves, or secondary to contamination of the lipid product, resulting in either local or systemic infection and venous irritation (6, 7, 9). In humans, subacute and delayed reactions have also been reported and include fat overload syndrome, which can result in fat embolism, hyperlipidemia, hepatomegaly, icterus, splenomegaly, thrombocytopenia, prolonged clotting times, and hemolysis (1, 6, 7). Hyperlipidemia is an unavoidable consequence of IVLE use and there is concern for precipitating pancreatitis in animals, even though it is yet to be proven in the acute setting with IVLE therapy. Numerous veterinary reports of successful treatment with IVLE for lidocaine, naproxen, carprofen, ibuprofen, vitamin D, bromethalin, and several other toxicoses have been published (11–18).

The decision to use IVLE in this patient was mainly due to the potential of a relatively high concentration of toxin ingestion, the extended-release nature of the drug, and presence of clinical signs. The owners also reported limited finances and were unable to afford lengthy hospitalization. The expectation of using IVLE was to accelerate the metabolism of the amphetamine in hopes of a shorter hospitalization time and thereby lower financial cost.

Human dosage recommendations are to use a 20% IVLE at: 1.5 ml/kg IV bolus, followed by a CRI of 0.25 ml/kg/min (6, 7, 10). The use of a filter is also recommended, in order to prevent infusion or larger lipid droplets (7). Currently in veterinary medicine, there is no established recommended dose for IVLE administration in the treatment of lipophilic drug toxicosis, although many published reports use this dose (4, 8, 9).

In humans, amphetamines are well-absorbed orally with peak plasma levels occurring 1–3 h after ingestion and with Adderall XR, maximum plasma concentrations occur about 7 h after ingestion (19). In this case report, the presentation occurred no later than 3 h from presentation, and it would be expected that the amphetamine concentration would continue to increase in a linear fashion. Albeit, a limitation is that this is based on data obtained from human pharmacokinetic studies (19). Additional unknown factors and limitations are whether the pills were crushed or chewed by the patient, that could theoretically increase the rate of absorption. A confounding factor was that other treatments such as IV fluids and sedatives were used. Despite documented lower amphetamine concentrations 1 h after IVLE therapy, there was a recurrence of clinical signs noted 4 h later, and it would have been interesting to measure concentrations at additional time points. Lastly, it is unknown if lipemia would interfere with the assay analysis of the amphetamine.

**Figure 2:**
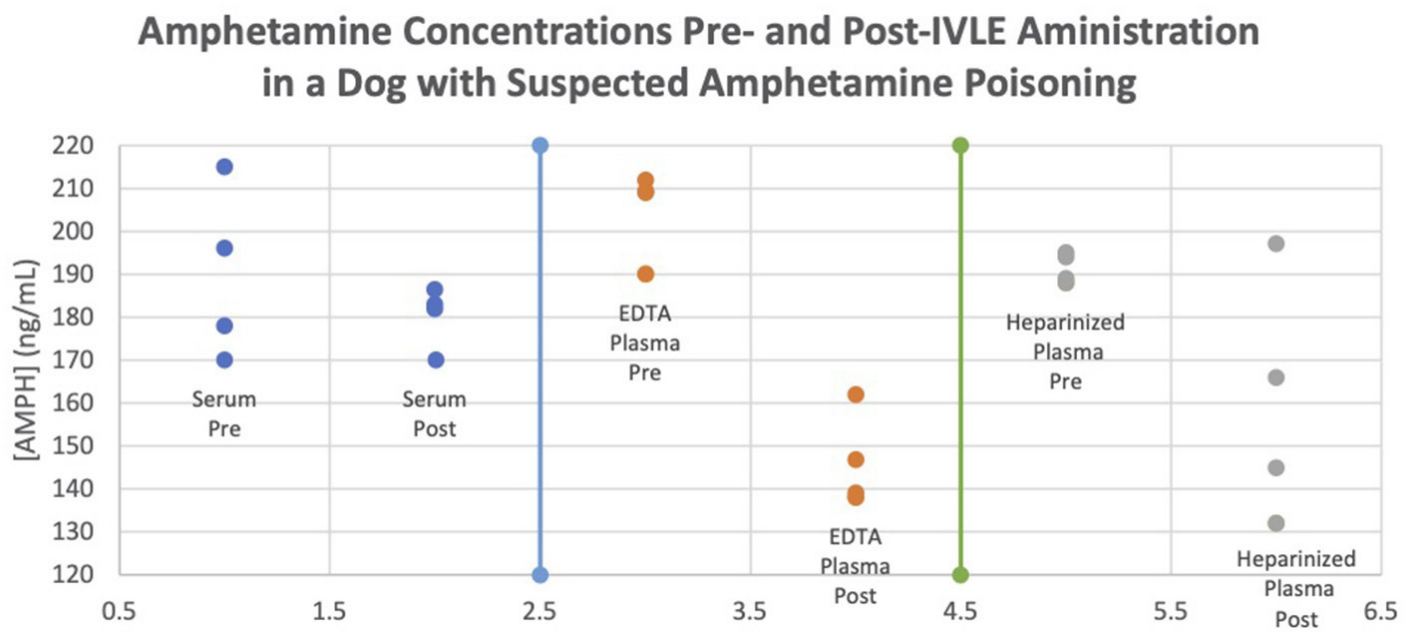
Amphetamine concentrations pre- and post-IVLE administration in a dog with suspected amphetamine poisoning.

To detect any *in vitro* differences caused by anticoagulants, the amphetamine blood samples were measured in heparinized plasma, EDTA plasma, and serum. There were differences noted amongst the tube samples, with the smallest difference noted in serum and the greatest in EDTA plasma (Figure 2). It is noteworthy that in this report, the analysis of amphetamine concentrations with heparinized plasma did not change as much as that of the EDTA plasma. This is most likely due to lipemia, which is known to interfere with some assays due to either light-scattering effects or binding to the analyte. There have been cases of interference between heparin and some immunoassays but the effects on this immunoassay are unknown. Further studies are warranted evaluating the effects of IVLE therapy and sampling with different anticoagulants and to determine how a decreased amphetamine concentration correlates with clinical signs. This is additionally important as there are many different drug formulations of amphetamines, which could affect the decision to use IVLE.

## Concluding Remarks

The authors propose that IVLE was responsible for the rapid rate of reduction in plasma and serum amphetamine concentration in this patient, with plasma concentration being more notable than that of serum. Samples were taken immediately prior to IVLE administration and 1 h post-IVLE therapy, with no other interventions administered during this interval. Additionally, because amphetamine has a long half-life in dogs of around 12 h (longer with extended-release formulas, such as in this case), drug concentrations would not have been expected to decline to the concentrations reported at these times (1, 3, 19). Questions that remain include why heparinized plasma is affected to a greater degree than either the EDTA plasma or serum samples and why some serum samples were higher post-IVLE (Figure 2). With the understanding of why we think IVLE therapy works, and this first known veterinary documentation of amphetamine levels before and after IVLE therapy, these results may prompt a veterinary practitioner to utilize IVLE therapy in cases of amphetamine overdose, and it is suggested that this potentially could shorten hospitalization length and cost.

## Data Availability Statement

The raw data supporting the conclusions of this article will be made available by the authors, without undue reservation.

## Author Contributions

SH created the original draft. MM provided edits. DB and RH analyzed samples. All authors contributed to manuscript revision, read, and approved the submitted version.

## Conflict of Interest

The authors declare that the research was conducted in the absence of any commercial or financial relationships that could be construed as a potential conflict of interest. The reviewer JH declared a past co-authorship with one of the authors MM to the handling editor.

## Publisher's Note

All claims expressed in this article are solely those of the authors and do not necessarily represent those of their affiliated organizations, or those of the publisher, the editors and the reviewers. Any product that may be evaluated in this article, or claim that may be made by its manufacturer, is not guaranteed or endorsed by the publisher.
